# Differences in ankle and knee muscle architecture and plantar pressure distribution among women with knee osteoarthritis

**DOI:** 10.1002/jfa2.12028

**Published:** 2024-05-31

**Authors:** Nazli Busra Cigercioglu, Zilan Bazancir‐Apaydin, Hakan Apaydin, Gul Baltaci, Hande Guney‐Deniz

**Affiliations:** ^1^ Faculty of Physical Therapy and Rehabilitation Department of Musculoskeletal Physiotherapy and Rehabilitation Hacettepe University Ankara Turkey; ^2^ Faculty of Health Science Department of Physiotherapy and Rehabilitation Ankara Medipol University Ankara Turkey; ^3^ Department of Rheumatology Ankara Etlik City Hospital Ankara Turkey; ^4^ Department of Physiotherapy and Rehabilitation Istanbul Atlas University Faculty of Health Science Istanbul Turkey

**Keywords:** foot, knee osteoarthritis, muscle architecture, muscles, ultrasonography

## Abstract

**Background:**

The aim of this study was to compare the plantar pressure distribution and knee and ankle muscle architecture in women with and without knee osteoarthritis (OA).

**Methods:**

Fifty women with knee OA (mean age = 52.11 ± 4.96 years, mean Body mass index (BMI) = 30.94 ± 4.23 kg/m^2^) and 50 healthy women as a control group (mean age = 50.93 ± 3.78 years, mean BMI = 29.06 ± 4.82 kg/m^2^) were included in the study. Ultrasonography was used to evaluate knee and ankle muscles architecture and femoral cartilage thickness. The plantar pressure distribution was evaluated using the Digital Biometry Scanning System and Milleri software (DIASU, Italy). Static foot posture was evaluated using the Foot Posture Index (FPI), and pain severity was assessed using the Visual Analog Scale.

**Results:**

The OA group exhibited lower muscle thickness in Rectus Femoris (RF) (*p* = 0.003), Vastus Medialis (VM) (*p* = 0.004), Vastus Lateralis (*p* = 0.023), and Peroneus Longus (*p* = 0.002), as well as lower Medial Gastrocnemius pennation angle (*p* = 0.049) and higher Fat thickness (FT) in RF (*p* = 0.033) and VM (*p* = 0.037) compared to the control group. The OA group showed thinner femoral cartilage thickness (*p* = 0.001) and higher pain severity (*p* = 0.001) than the control groups. FPI scores were higher (*p* = 0.001) in OA group compared to the control group. The plantar pressure distribution results indicated an increase in total surface (*p* = 0.027), total load (*p* = 0.002), medial load (*p* = 0.005), and lateral load (*p* = 0.002) on dominant side in OA group compared to the control group.

**Conclusions:**

Knee and ankle muscle architecture, knee extensor muscle FT, and plantar pressure distribution in the dominant foot differed in individuals with knee OA compared to the control group.

## 1 INTRODUCTION

Knee osteoarthritis (OA) is a chronic degenerative joint disease that leads to the degradation of articular cartilage, chronic joint pain, joint stiffness, and muscle weakness [1]. In OA, femoral cartilage thickness decreases [2, 3], and knee pain during daily activities increases, accompanied by a decrease in knee extension strength [4]. The presence of OA is associated with early muscular changes and appears to exacerbate the changes in thigh muscles that are similar to those observed during the aging process [5]. Muscle weakness, especially in quadriceps muscle, is commonly reported in individuals with OA and is often the first sign detected [6]. There is also evidence suggesting that muscle weakness is linked to unfavorable changes in muscle architecture parameters, including a reduction in muscle thickness and fascicle length in individuals with OA [7, 8].

The muscle architecture parameters such as muscle thickness, pennation angle, and fascicle length are considered to be associated with muscle strength [9]. Ultrasound imaging, a noninvasive and safe method, can easily assess these parameters and provide reliable information on skeletal muscles [10]. Moreover, several studies have demonstrated significant correlations between ultrasound imaging of quadriceps femoris (QF) thickness, pennation angle, and fascicle length with isometric and isokinetic strength of knee extensors [11, 12, 13, 14].

Pennation angle is closely linked to the maximal force capacity of a muscle fiber, as it correlates with the number of sarcomeres arranged in parallel [15]. Fascicle length is proportional to maximum contraction velocity, as it relates to the number of sarcomeres arranged in series [16]. Muscle thickness correlates with muscle cross‐sectional area and, therefore, muscle strength [12, 14]. Previous studies have demonstrated disrupted knee muscle architecture in individuals with OA, resulting in shorter Vastus Lateralis (VL) fascicle length and lower Vastus Medialis (VM) muscle thickness [5, 8, 17]. However, there is no consensus on the architectural characteristics of lower extremity muscles in individuals with OA. Examining the differences in lower extremity muscle architecture in OA could provide insights into factors such as pain inhibition and potential chronic low‐grade inflammation.

Similar to muscle architecture, foot pressure distribution is changed in individuals with OA due to increased pronation of the subtalar joint, foot arch collapse, and pes planus [18, 19, 20]. Discerning between foot posture changes that contribute to OA and those resulting from the compensatory changes because of OA itself is challenging. Abnormalities in foot posture could potentially trigger the onset of OA. Conversely, as OA progresses, compensatory changes may occur in foot posture as a mechanism to adapt [21]. Abnormal rotation of the tibia and femur caused by genu varum or varus in individuals with OA is thought to be associated with biomechanical changes in the subtalar joint motion [19, 20]. These alterations in mechanical alignment of the lower extremity are associated with foot pressure distribution [18, 22, 23]. In individuals with OA, it is reasonable to expect that changes in the functional use of lower extremity muscles, which result from alterations in knee and foot biomechanics, could indicate alterations in ankle muscle architecture in addition to knee muscles. However, there is limited research assessing these differences in muscle architecture and foot pressure distribution among individuals with OA.

Therefore, the aim of the present study was to compare the plantar pressure distribution and knee and ankle muscle architecture in women with and without OA. Only women with OA were included in the study to establish a homogeneous group. Our primary hypothesis was that women with OA have early changes in both knee and ankle muscle architecture, characterized by a reduction in pennation angle, fascicle length, and muscle thickness. Second hypothesis was that women with OA would exhibit altered foot pressure distribution, characterized by an increase in total loading compared to age‐matched healthy women.

## 2 MATERIALS & METHODS

### 2.1 Study design

This comparative cross‐sectional study was approved by the Institutional Ethics Review Board of the authors' affiliated institutions **(Ethics Board of Hacettepe University, ID. GO 22/290)**. Written informed consent was obtained from all individuals, and all procedures were conducted in accordance with the Declaration of Helsinki. The data were collected between March 2021 and December 2022.

### 2.2 Participants

The study included 50 female individuals with knee OA as the OA group and 50 age‐ and body mass index (BMI)‐matched healthy women as the control group. Following medical examination, radiologic imaging was requested from individuals with suspected OA according to the examination findings (using medical history and clinical examination: 1‐age >50 years, 2‐ morning stiffness<30 min, 3‐crepitus on active motions, 4‐bony tenderness, 5‐bony enlargement, and 6‐no palpable warmth of synovium) [24]. All women with OA exhibited apparent radiographic changes in the bilateral or unilateral knees indicative of OA and were diagnosed as Kellgren Lawrence (K‐L) grade 2. The inclusion criteria for the women with OA were aged 45–65 years, experiencing activity‐related knee pain, having morning knee stiffness lasting for at least 30 min, living independently, and experiencing knee pain on most days for 3 months or more. The exclusion criteria for OA were having lower extremity surgery, congenital hip dislocation or developmental conditions affecting the lower limb, systemic inflammatory arthritis, polyneuropathy/lower extremity neuropathy, severe radiculopathy, having undergone physical therapy, exercises or knee injections for the knee in the previous 6 months, congenital foot deformity, use of gait devices, and BMI higher than 35 kg/m^2^.

Healthy women who were age‐ and BMI‐matched were included in the control group. Inclusion criteria for the control group were women aged 45–65 years, ability to live independently, ability to walk without assistive devices, no self‐reported history of knee pain, and absence of clinical OA symptoms or signs. Radiologic evaluation was not performed in the control group due to the absence of OA symptoms or signs. The control group was excluded if they had any knee or lower extremity injury or surgery, pain around the lower extremity and BMI higher than 35 kg/m^2^.

### 2.3 Measurements

***Pain severity***: The Visual Analog Scale was used to evaluate the severity of knee and foot pain during activity [21]. The 10‐cm horizontal line was defined as 0 ″no pain” and 10 ″very severe pain”, and the individuals were asked to indicate their knee and foot pain level by drawing a line. This line was then measured in centimeters on both the dominant and nondominant side [25].

***Static foot posture***: The Foot Posture Index (FPI) was used to evaluate foot posture while standing in a relaxed position. The FPI has been demonstrated to be a valid and reliable tool in individuals with OA [26]. The FPI consisted of six items: (1) palpation of the talar head, (2) assessment of the curvature of the supra and infra lateral malleolus, (3) evaluation of the frontal plane position of the calcaneus, (4) identification of any prominence in the region of the talonavicular joint, (5) examination of the congruence of the medial longitudinal arch, and (6) measurement of abduction/adduction of the forefoot on the rear foot. Each item was scored from −2 to +2, with a total score ranging from −12 to +12. A higher positive score indicated a more pronated foot. A score of ≥6 indicated a pronated foot type, while scores of 0–5 indicated a neutral foot type, and scores of ≤ ‐1 indicated a supinated foot type [27, 28]. The OA side and the dominant side were assessed. All static foot posture data were collected by a physiotherapist with 5 years of experience in foot assessment (NBC). To avoid bias, the physiotherapist performing the assessment was blinded to the participants' group assignments. Limb dominance was determined by asking all participants which leg they would use to kick a ball [29].

***Muscle architecture***: Ultrasound, a measurement method with demonstrated validity and reliability in individuals with OA [30, 31], was used to evaluate the knee and ankle muscles. A total of six muscles were evaluated, including three knee muscles and three ankle muscles: Rectus Femoris (RF), VM, VL, Tibialis Anterior (TA), Peroneus Longus (PL), and Medial Gastrocnemius (MG). Muscle thickness, pennation angle, fascicle length, and Fat thickness (FT) of each muscle were evaluated using B‐Mode ultrasonography (Esaote MyLab X8 eXP Ultrasound System, Florence, Italy) and a linear array transducer (4–11.4 MHz). Given the noted muscle weakness in the muscles around the knee in OA, and in alignment with our hypothesis, we hypothesized that there may be differences in the muscles around the foot in these patients. Therefore, we chose to evaluate these muscles. All data on muscle architecture were collected by the same rheumatologist with 5 years of experience in ultrasound assessment **(HA).**

The images of the RF, VM, VL, and TA muscles were obtained with participants in a supine position [5, 17, 32, 33, 34, 35]. For the MG and PL muscle images, participants were instructed to lie prone [33, 34, 36]. To enhance reproducibility and minimize the risk of sampling a muscle obliquely, the transducer was oriented parallel to the muscle fascicles and perpendicular to the skin [5].

**FIGURE 1:**
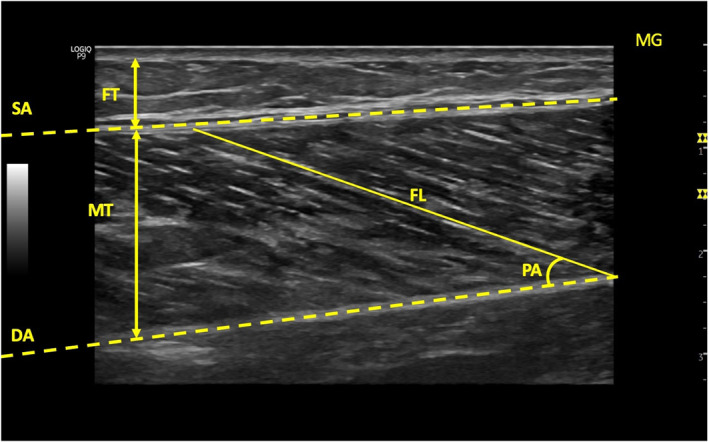
Ultrasound images of the medial gastrocnemius. DA, deep aponeurosis, FL, fascicle length, FT, fat thickness, MG, medial gastrocnemius, MT, muscle thickness, PA, pennation angle, SA, superficial aponeurosis.

The image was considered optimized when a thin layer of gel was visible between the skin and the transducer, signifying that no manual compressive forces were distorting the muscle after identification. The same researcher **(HA)** then slightly retracted the transducer to avoid compressing the muscle. Ultrasound images were analyzed using Image J program (Version 1.53p, 2022, National Institutes of Health, Bethesda, MD, USA) by the same researcher **(NBC)** (Figure 1). The same procedure was employed for each muscle group, with measurements taken for both muscle architecture and FT. After completing all images from the first extremity, the identical technique was utilized to image the opposite lower extremity. Muscle thickness was measured as the distance between the internal borders of the superficial and deep aponeuroses. FT of all muscles was measured as the distance between the skin of the superficial aponeuroses [35]. Pennation angle was defined as the angle between the muscle fascicle line and the deep aponeuroses. Fascicle length was defined as the distance between the origin of the fascicle at the superficial aponeuroses and insertion of the same fascicle in the deep aponeuroses [5].

***Femoral cartilage thickness***: To determine femoral cartilage thickness, individuals assumed a supine position with their knees comfortably flexed to the maximum extent. The probe was positioned axially on the suprapatellar region. Femoral cartilage thickness was assessed at three discrete points on both knees: specifically, at the levels of the lateral condyle, intercondylar area, and medial condyle [15]. Water‐soluble gel was applied between the transducer and the skin to support acoustic coupling, without applying pressure to the muscle. The transducer was oriented axially toward outer edge of the patella. In addition, femoral cartilage thickness measurement is a valid and reliable method [37].

***Plantar pressure distribution***: Plantar pressure distribution was performed using the Digital Biometric Images Scanning System and relevant Milleri software (Diagnostic Support; Diasu Health Technologies) [38]. The platform employed in this study consisted of a 5‐m long and 40‐cm‐wide walkway equipped with 4024 sensors capable of sampling data at a frequency of 300 MHz. The force platform assessed plantar pressure on both feet while standing and calculated the average percentage of pressure distribution for each foot. Participants were instructed to stand barefoot on the platform for 10 s, facing a reference point, with their arms hanging comfortably by their sides. During these measurements, the individuals were instructed not to consciously adjust their posture [39].

**FIGURE 2:**
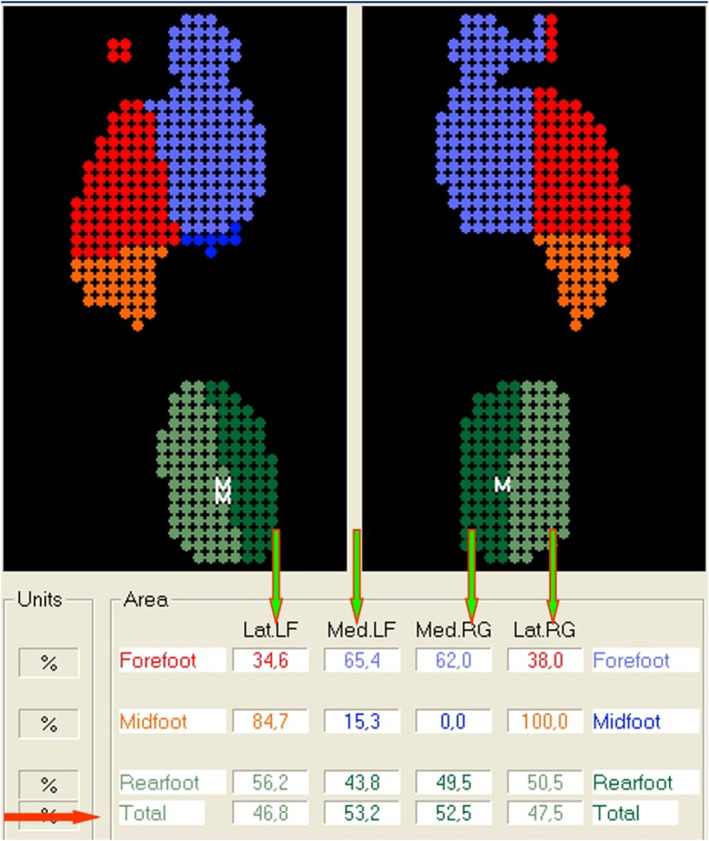
Analysis results of the plantar pressure distribution. P. Avg, average pressure, Pmax, maximum pressure.

The static evaluation provided information on numerical surface and loading values, both globally (for each foot) and partially (relative to the rearfoot, forefoot, medial and lateral load). Recorded parameters included maximum foot pressure (FP_max_), average foot pressure (Pavg.), total surface area, forefoot load (FFL), rearfoot load (RFL), total load on the foot, medial load, lateral load, foot angle (FA), and foot progression angle (FPA) of the women (Figure 2). FA is defined as the angle between the direction of progression of the individual and a reference line on the sole of each foot [40]. FPA is the angle between the longitudinal foot axis and the vertical axis of the foot [41].

The plantar pressure distribution outcomes of the related knee in patients with unilateral involvement and the most symptomatic knee in patients with bilateral knee involvement were compared with matched knees of the controls [39]. Plantar pressure distribution was performed by a physiotherapist with 7 years of experience in this field **(NBC).** To avoid bias, the physiotherapist conducting the assessment was blinded to the participants' group assignments.

### 2.4 Statistical analysis

The statistical analysis was performed using the Statistical Product and Service Solutions (SPSS) Statistics software package (Version 23.0, SPSS Inc., Chicago). The normality of the distribution of the data was assessed with visual and analytical methods using histograms, Q‐Q plots, and Kolmogorov Smirnov tests. For the comparison analysis, the affected side of the patients and the corresponding side of the controls were used. The pain levels, foot posture, ultrasonography data, and plantar pressure distribution were normally distributed. Therefore, the data among the groups were compared using an independent samples *t*‐test. The Levene test was used to assess the homogeneity of the variances. An overall *p* value of less than 0.05 was considered to show a statistically significant result.

A priori sample size calculation was conducted with G Power 3.1.9.2 (Franz Faul, University of Kiel). In the power analysis in which muscle thickness was taken as the main criterion and calculations were made, it was found that the sample size of at least 45 people for each group had a power of 0.80, an effect size of 0.40, and an alpha value of 0.05 [5].

## 3 RESULTS

All data were found to be normally distributed, and demographic and clinical characteristics were similar between groups (*p* > 0.05). Knee pain (*p* = 0.001) and foot pain severity (*p* = 0.001) as well as FPI were higher in the OA group compared to the control groups (*p* = 0.001) (Table 1).

**TABLE 1:** Demographic and clinical characteristics of the OA and control groups.

Characteristics	OA group (*n* = 50)	Control group (*n* = 50)	*p*
Age (year)	52.11 ± 4.96	50.93 ± 3.78	0.816
Gender, *n* (%)	50 (100)	50 (100)	1.000
Female			
BMI (kg/m^2^)	30.94 ± 4.23	29.06 ± 4.82	0.734
KL scores, *n* (%)	50 (100)	0 (100)	**0.001** ^a^
Grade 2			
Affected side, *n* (%)		‐	‐
Unilateral	42 (84)		
Bilateral	8 (16)		
Knee pain (VAS), mean ± SD			
Dominant side	4.53 ± 2.29	0	**0.001** ^a^
Non‐dominant side	3.81 ± 2.58	0	**0.001** ^a^
Foot pain (cm), mean ± SD			
Dominant side	0.92 ± 0.35	0	**0.001** ^a^
Non‐dominant	0.83 ± 0.45	0	**0.001** ^a^
FPI (point), mean ± SD			
Dominant side	4.14 ± 2.67	2.00 ± 1.44	**0.001** ^a^
Nondominant side	3.80 ± 2.63	2.07 ± 2.2	**0.001** ^a^
Foot posture, *n* (%)			**0.009** ^a^
Neutral foot	33 (66)	46 (92)	
Pronated foot	15 (30)	2 (4)	
Supinated foot	2 (4)	2 (4)	

Abbreviations: BMI, Body mass index; FPI, foot posture index; KL, Kellgren Lawrence; OA, osteoarthritis; SD, standard deviation; VAS, visual analog scale.

^a^ Independent samples *t*‐test.

The OA group displayed lower muscle thickness in the RF (*p* = 0.003), VM (*p* = 0.004), VL (*p* = 0.023), and PL (*p* = 0.002) muscles, as well as a lower MG pennation angle (*p* = 0.049), and higher FT in the RF (*p* = 0.033) and VM (*p* = 0.037), compared to the control group (Table 2). Moreover, a significant difference in femoral cartilage thickness was found between the groups (*p* = 0.001) (Table 3).

**TABLE 2:** Muscle architecture and fat thickness results of the OA and control groups.

	Muscle thickness			Pennation angle			Fiber length			Fat thickness			
	OA group	Control group	*p*	OA group	Control group	*p*	OA group	Control group		OA group	Control group	*p*
RF	1.76 ± 0.49	2.06 ± 0.26	**0.003** ^a^	12.33 ± 2.31	11.84 ± 3.02	0.856	7.86 ± 0.76	7.16 ± 1.06	0.991	1.47 ± 0.45	1.32 ± 0.19	**0.033** ^a^
VM	1.62 ± 0.25	1.64 ± 0.20	**0.004** ^a^	14.27 ± 4.74	13.62 ± 2.2	**0.008** ^a^	4.73 ± 0.86	5.94 ± 1.52	0.273	1.32 ± 0.63	1.14 ± 0.28	**0.037** ^a^
VL	1.82 ± 0.44	2.1 ± 0.45	**0.023** ^a^	19.3 ± 4.25	15.2 ± 1.72	0.688	6.93 ± 0.57	6.06 ± 1.43	0.930	1.86 ± 0.72	1.52 ± 0.19	0.325
TA	1.86 ± 0.23	0.88 ± 0.19	0.096	12.33 ± 2.75	12.26 ± 2.38	0.903	4.36 ± 0.37	3.72 ± 0.46	0.584	‐	‐	‐
PL	0.62 + 0.15	0.64 + 0.18	**0.002** ^a^	10.5 ± 0.6	8.46 ± 1.41	0.786	2.48 ± 0.92	2.54 ± 0.96	0.741	‐	‐	‐
MG	2.03 ± 0.15	1.9 ± 0.22	0.956	21.97 + 1.89	25.38 + 2.59	**0.049** ^a^	2.83 ± 0.41	3.94 ± 0.53	0.605	1.3 ± 0.34	1 ± 0.27	0.151

Abbreviations: MG, medial gastrocnemius; OA, osteoarthritis; PL, peroneus longus; RF, rectus femoris; TA, tibialis anterior; VL, vastus lateralis; VM, vastus medialis.

^a^ Independent samples *t* test.

**TABLE 3:** Femoral cartilage thickness results of the OA and control groups.

Parameters	OA group (*n* = 50)	Control group (*n* = 50)	*p*
Femoral cartilage medial	1.80 ± 0.33	2.34 ± 0.32	**0.001** ^a^
Femoral cartilage intercondylar area	2.22 ± 0.41	3.44 ± 0.28	**0.001** ^a^
Femoral cartilage lateral	1.95 ± 0.12	2.66 ± 0.42	**0.001** ^a^

Abbreviation: OA: osteoarthritis.

^a^ Independent samples *t* test.

The results of plantar pressure distribution analysis revealed a significant difference in total surface area between the OA group and the control group (*p* = 0.027). The OA group demonstrated higher total load (*p* = 0.002), medial load (*p* = 0.005), and lateral load (*p* = 0.002) on the dominant side compared to the control group. Furthermore, the OA group exhibited a lower FA on the nondominant side compared to the control group (*p* = 0.016). However, no significant differences were observed in FPmax (*p* = 0.934), Pavg (*p* = 0.517), FFL on the dominant side (*p* = 0.257), and RFL (*p* = 0.138) between the groups (Table 4).

**TABLE 4:** Plantar pressure distribution results of the OA and control groups.

Parameters	OA group (*n* = 50)	Control group (*n* = 50)	*p*
Total surface (cm^2^)	260.84 ± 41.87	234.22 ± 55.70	0.150
FP_max_ (g/cm^2^)	557.79 ± 186.12	531.43 ± 105.51	0.934
P avg. (g/cm^2^)	300.41 ± 47.38	302.27 ± 88.64	0.517
Total surface (cm^2^)			
Dominant side	122.08 ± 26.17	108.65 ± 33.57	**0.027** ^a^
Nondominant side	129.24 ± 21.81	118.40 ± 31.10	0.203
Foot angle (°)			
Dominant side	8.54 ± 3.69	10.58 ± 3.17	0.195
Non‐dominant side	9.31 ± 3.63	11.06 ± 3.41	**0.016** ^a^
Foot progression angle (°)			
Dominant side	10.74 ± 6.28	11.31 ± 5.69	0.306
Non‐dominant side	9.13 ± 6.19	8.46 ± 3.83	0.475
Dominant side			
Total load (kg)	37.72 ± 8.11	32.40 ± 8.09	**0.002** ^a^
Medial load (kg)	18.35 ± 4.03	16.04 ± 3.9	**0.005** ^a^
Lateral load (kg)	19.3 ± 4.43	16.41 ± 4.34	**0.002** ^a^
Dominant side			
Forefoot load (kg)	23.07 ± 5.35	23 ± 10.89	0.257
Rearfoot load (kg)	27.57 ± 6.28	27.08 ± 6.31	0.138

Abbreviations: FPmax, maximum pressure; OA, osteoarthritis; Pavg, average pressure.

^a^ Independent samples *t*‐test.

## 4 DISCUSSION

The findings of this study indicated that women with OA exhibited plantar pressure distribution and knee and ankle muscle architecture differences. In particular, the OA group showed a decrease in RF, VM, VL, and PL muscle thickness, as well as a lower pennation angle of the MG muscle and increased FT of the RF and VM muscles compared to control group. Moreover, individuals with OA demonstrated increased total and mediolateral loading in the dominant foot compared to the control group.

Previous studies have consistently demonstrated individuals with OA experience changes in foot posture and increased pain levels [21, 25, 42]. Specifically, these patients exhibit a higher prevalence of foot pronation and a prone foot position, which are commonly associated with OA [42, 43]. Furthermore, Al‐Bayati et al. [44] revealed a correlation between varus alignment in OA and pronated foot posture, while the clinical severity of OA is linked to a supinated foot posture. Akaltun et al. [21] reported a distribution of 16.5% supinated foot, 73% neutral foot, and 10.5% pronated foot in OA patients, compared to 6.5% supinated foot, 89.6% neutral foot, and 3.9% pronated foot in control group. The prevalence of neutral foot posture was lower, whereas supinated and pronated foot postures were more prevalent in patients with OA [21]. Authors elucidated that abnormalities in foot posture can result in alterations in force distribution throughout the lower extremity, including the knee joint. Moreover, abnormal foot posture may contribute to the development of OA, and advanced OA can subsequently induce changes in foot posture as a compensatory mechanism [21]. Consistent with prior studies [21, 42, 43, 44], the present study observed a higher rate of pronated foot posture (32%) in OA group compared to control group (4.7%). Furthermore, the current study identified higher knee and foot pain levels and an increased prevalence of foot pronation in OA group compared to control group. These findings further support the notion that alterations in foot biomechanics may contribute to mild foot pain and potentially serve as a risk factor for OA, with foot postures varying according to the affected knee compartment. Additionally, the presence of a pronated foot posture in OA group may impact plantar loading. Future research should focus on utilizing advanced biomechanical measurements and dynamic plantar pressure analysis to gain a deeper understanding of these relationships.

Aily et al. [5] conducted an evaluation of VL muscle architecture and strength in patients with OA, categorizing them into middle‐aged and older groups. Their findings demonstrated that the patients with OA exhibited the smallest muscle architecture parameters and displayed the lowest isometric and concentric peak torques when compared to the control group. Similarly, Vaz et al. [8] observed that women with OA had weaker and smaller muscles, as well as shorter VL fascicle length compared to age‐matched healthy individuals. They proposed that these differences could be attributed to a reduction in QF muscle mass, along with decreases in muscle thickness and cross‐sectional area [8]. Smaller fascicle length, as reflected by the pennation angle, may explain these findings, as muscle atrophy is occasionally associated with a decrease in pennation angle [17]. Taniguchi et al. [18] reported significantly lower muscle thickness in the VM and Vastus Intermedius (VIM), as well as larger echo intensity of the VM, VIM, TA, and gluteus medius in patients with OA. Another study indicated a 12% reduction in cross‐sectional area of the QF is in OA patients [45]. Specifically, muscle atrophy in the VM may be more prevalent compared to other muscles of the QF.

Muscle architecture is influenced by various factors, including age, strength, pain, and postural adaptations [25, 45]. For instance, Ikeda et al. [45] observed that older groups exhibited smaller QF cross‐sectional areas and lower intermuscular density, suggesting that age‐related atrophy of the quadriceps‐dominant muscles may influence the pathophysiology of OA. Consistent with the previous research [5, 8, 17, 45], the present study revealed reduced muscle thickness in the RF, VM, VL, and PL, as well as a decrease in the pennation angle of MG in OA patients compared to the control group. Furthermore, it appears that the muscle architecture of ankle plantar flexors and evertors is also altered in OA. The diminished pennation angle of the MG may indicate muscle atrophy, aligning with findings from studies conducted by Blazevich et al. [46] and Ikeda et al. [45]. Additionally, the noticeable decrease in PL muscle thickness may be related to altered foot biomechanics. Previous research has demonstrated that females with a pronated foot posture exhibit smaller muscle thickness in the RF and VM oblique compared to those with a normal foot posture [42]. In light of these findings, clinicians should consider changes in ankle plantar flexor and evertor muscle architecture and consider individualized gait modifications, footwear, and foot orthoses.

Previous studies have consistently reported higher FT in RF and VM muscles among individuals with OA [47, 48, 49]. Specifically, one study indicated significantly greater intramuscular fat in the QF and hamstring muscles, excluding the VL, in OA individuals compared to healthy individuals [49]. Similarly, other studies demonstrated higher levels of intramuscular fat in the quadriceps and hamstring muscles of OA individuals [47, 48]. The findings of the present study align with these previous investigations [47, 48, 49], as we observed elevated FT in the RF and VM muscles in OA individuals compared to the control group. It is plausible that alterations in muscle architecture contribute to increased FT in the QF muscle. There is an established inverse relationship between a muscle's capacity to generate force and the extent of muscular fat infiltration [21]. Despite the similar BMI between the OA and control groups, the higher FT observed in OA individuals is likely associated with changes in muscle architecture, reduced muscle strength, and disuse due to pain.

The present study further supports existing evidence regarding the characteristic loss of hyaline cartilage in women with OA. Malas et al. [15] assessed femoral cartilage thickness at multiple levels, including the lateral condyle, intercondylar area, and medial condyle, and observed significantly reduced cartilage thickness at all three locations compared to healthy individuals. Similarly, Tuna et al. [2] established a positive correlation between femoral cartilage thickness and isometric strength values at 30° and isokinetic work values at 180°/s. Consistent with previous findings, the present study confirmed thinner hyaline cartilage at the lateral condyle, intercondylar area, and medial condyle levels in OA group compared to the control group.

Individuals with OA exhibit distinct characteristics in their foot pressure distribution compared to healthy individuals, as evidenced by previous research [50, 51, 52]. Notably, individuals with both pes planus and OA demonstrate higher peak pressure in specific regions such as the second metatarsophalangeal joint, hallux, and second toe [50]. Moreover, a study identified greater maximum force in the medial midfoot of planus feet compared to normal and cavus feet [51]. Another investigation reported potential differences in the center of pressure during medial foot shift, which could lead to an increase in the knee adduction moment arm [43]. Findings of the present study suggested that individuals with OA adopt strategies to reduce loading on the knee joint while walking. Consistent with previous findings, the present study revealed higher total, medial, and lateral loading in the dominant foot of OA individuals compared to control group. Furthermore, individuals with OA exhibited a decreased FA in the nondominant foot, indicating distinct foot adaptations. These results diverge from the findings of Akaltun's study [21], which primarily observed pes planus in their sample. On the other hand, the present study revealed a 7.5 times higher prevalence of prone foot posture in the OA group compared to the control group, even though the prevalence of neutral posture was high in the OA group. Consequently, this study identified excessive lateral loading alongside increased total and medial loading. Therefore, this study showed that medial loading increased while lateral loading continued; this may be due to the higher proportion of pronated foot posture individuals in the sample group. To gain further insights, future investigations should explore the impact of varying foot postures on thigh and leg muscle architecture, as well as plantar pressure distribution, in individuals with OA.

This study has several limitations. Firstly, it is important to note that the sample of this study consisted solely middle‐aged women with K‐L grade 2, which limits the generalizability of the findings to all individuals with OA. Gender is a known risk factor for OA, higher incidence rates in women compared to men [53]. To standardize the group, only women were included in the study. However, this homogenization can also be viewed as a strength. Another limitation was that we considered evaluating the tibialis posterior muscle at the beginning of the study, but we excluded this muscle evaluation because we could not get a clear ultrasound image of the muscle. Secondly, no radiologic evaluation was performed in the control group. Radiologic evaluation was not deemed necessary because of the absence of OA symptoms or signs in these subjects and the risk of radiation. Lastly, the assessment of foot characteristics was conducted in a static position. Incorporating dynamic analysis during gait would have provided valuable clinical insights. The literature lacks studies exploring the relationship between plantar pressure distribution, ankle muscle architecture, and OA. Nonetheless, the present study contributes to our understanding of the observed differences in muscle architecture and plantar pressure distribution between women with OA and control group. This study is the first to comprehensively evaluate foot posture, plantar pressure distribution, and muscle architecture of the knee and ankle utilizing ultrasonography, thereby providing novel insights into this field. However, there remains a need for long‐term studies that comprehensively assess the lower extremity, including hip involvement and gait analysis.

In conclusion, our study provides evidence that knee and ankle muscle architecture, foot posture, and plantar pressure distribution differ in women with OA compared to controls. The differences in foot posture may affect the muscle architecture of the lower extremity or vice versa. The clinical significance of our study's findings lies in the recognition that OA affects not only knee muscles but also ankle muscles, coupled with alterations in foot posture. Furthermore, prescribing strengthening exercises becomes crucial to prevent muscle weakness and increased fat deposition. Moreover, biomechanical interventions, such as insoles, should be recommended after individual evaluation to address foot pronation and correct posture. Clinicians should consider these biomechanical and functional alternations when designing treatment interventions, including foot orthoses, appropriate footwear selection, or gait modification for individuals with OA.

## AUTHOR CONTRIBUTIONS

**Nazli Busra Cigercioglu**: Conceptualization; methodology; formal analysis; investigation; writing – original draft preparation. **Zilan Bazancir‐Apaydin**: Methodology; formal analysis; investigation; writing – original draft preparation; resources. **Hakan Apaydin**: Formal analysis; investigation; funding acquisition. **Gul Baltaci**: Funding acquisition; supervision. **Hande Guney‐Deniz**: Conceptualization; writing – review & editing; resources; supervision.

## CONFLICT OF INTEREST STATEMENT

Not applicable.

## ETHICS STATEMENT

This study was approved by Hacettepe University's Research Ethics Board. Writtenand verbal consent was obtained before and throughout any participationin this study. Project ID is GO‐ 22/290.

## Data Availability

The datasets used and/or analyzed during the current study are available from the corresponding author on reasonable request.
